# Production of Graphene/Inorganic Matrix Composites through the Sintering of Graphene Oxide Flakes Decorated with CuWO_4_·2H_2_O Nanoparticles

**DOI:** 10.1021/acsomega.3c00063

**Published:** 2023-04-02

**Authors:** Fei Lin, Yuzhen Zhou, Ruoyu Xu, Mingyu Zhou, Andrew M. Connolly, Robert J. Young, Ian A. Kinloch

**Affiliations:** † Department of Materials and the National Graphene Institute, University of Manchester, Manchester M13 9PL, U.K.; ‡ Department of Electrical Equipment & Material, Global Energy Interconnection Research Institute Europe GmbH, Berlin 10623, Germany

## Abstract

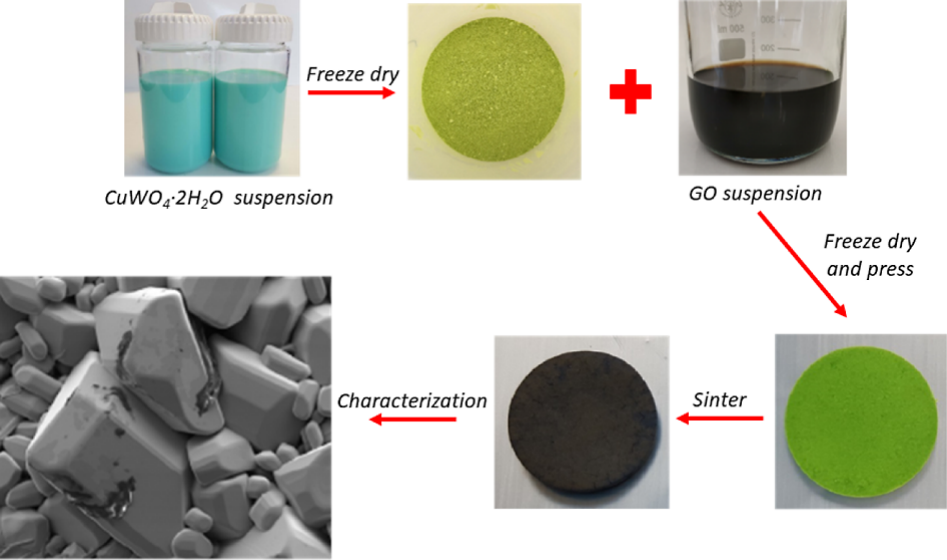

There is growing interest in graphene-reinforced inorganic matrix composites, but progress in this field is far behind that of polymer matrices due to difficulties in the processing of carbon materials in aggressive sintering environments, including oxidation and solubility in the host matrix. Copper–tungsten matrices are of particular interest in the power switching field but are difficult to produce due to the mutual insolubility of metals and poor wetting. Herein, composites were produced by decorating graphene oxide flakes with 8 nm diameter CuWO_4_·2H_2_O nanoparticles and then sintering them to form the final shape. The oxide nanoparticles were found to self-assemble into platelets on the surfaces of graphene flakes. Upon sintering, the presence of graphene was found to change the grain morphology from elongated needles to a polyhedral shape. It was found that, despite the nanosize of the CuWO_4_·2H_2_O particles used, the sintering conditions did not reduce the matrix to a pure metal; the sintered composites were found to be of mixed phase with copper tungstate and copper oxide present. Raman spectroscopy indicated that the graphene oxide became hydrogenated during the sintering process as a result of the reducing hydrogen atmosphere used.

## 1 Introduction

Copper–tungsten (Cu–W) composites combine the high melting point, high strength, and low thermal expansion coefficient of tungsten and maintain the high thermal and electrical conductivity of copper.^1^ Thus, Cu–W composites possess good electrical and thermal conductivities and are highly resistant to arc-erosion and oxidation. Unfortunately, the production of these composites is challenging due to both the mutual insolubility of the two metals and the high contact angle between solid tungsten and liquid copper. Thus, fully dense Cu–W composites can only be produced by powder metallurgy.^1^ Typically, Cu–W composites are manufactured industrially by the infiltration of liquid copper into porous tungsten skeletons.^2^ It is difficult, however, to make Cu–W composites with a uniform microstructure and thus high thermal and electrical conductivities, by such a method. Furthermore, this method is highly energy-intensive, expensive, and time-consuming. It also cannot make components with complex shapes. Alternative processing methods developed in the laboratory include spark plasma sintering (SPS),^3^ microwave sintering,^4^ and injection molding of bulk powders.^5^ These methods address some of the issues with infiltration, but the alloy structure is limited by the size of the starting powder. Attempts have been made in preparing CuW composites via electrodeposition, which have succeeded in depositing amorphous CuW structures upon a substrate.^6^ It is noted that electrodeposition results in the formation of Cu nanocrystals and a significantly lower W content than usually achieved via sintering.^1^ Another route to the preparation of Cu–W composites is by the reduction of copper tungstate (CuWO_4_). This materials is an n-type semiconductor with a band gap in the range 2.25–2.45 eV.^7^ Interest in CuWO_4_ has grown in recent years through its potential use in both photocatalytic^8−10^ and supercapacitor applications,^11,12^ sometimes in combination with carbon nanomaterials.^12,13^ Guo et al.^14^ recently produced CuWO_4_ and tungsten oxide (WO_3_) nanoparticles by a sol–gel process. These particles were then reduced in hydrogen and consolidated by SPS into giving a W70Cu30 composite. The nanoparticle-derived composites possessed a finer grain (350–400 nm) and a more homogeneous microstructure than those made by other methods, resulting in a higher relative density, higher thermal conductivity, and lower coefficient of thermal expansion, under optimized sintering conditions.

Cu–W materials are often used as high-voltage electrical contact parts, welding electrodes, fusion heat sinks,^15^ and thermal management devices.^1^ However, Cu–W electrical contact materials have a limited operating life of typically 20–30 strikes due mainly to the erosion from the high-voltage arcs. Recently, Dong et al.^16−19^ fabricated reduced graphene oxide (rGO)-reinforced W70Cu30 composites by the SPS of a mixture of bulk W powder, bulk Cu powder, and rGO. The rGO was found to improve the arc erosion properties and synergistically enhanced the electrical and thermal conductivity and mechanical properties of the Cu–W matrix. Indeed, graphene metal matrix composites (MMCs) are of increasing interest, but the progress in MMC-graphene is far behind that of polymer matrices due to the difficulties in processing carbon materials in aggressive sintering conditions, including oxidation and solubility in the host matrix.^20^ There have, however, been recent reports of producing copper^21^ and aluminum matrices with graphene-based reinforcements.^20,22,23^

Herein, we aim to produce precursors of Cu–W-rGO composites through the sintering of graphene oxide (GO) decorated with CuWO_4_·2H_2_O nanoparticles to combine the separate benefits discussed above of (i) using nanoparticle oxides to achieve a good Cu–W microstructure and (ii) addition of a graphene reinforcement. GO rather than graphene, was selected to enhance the interactions between the reinforcement and CuWO_4_·2H_2_O. The CuWO_4_·2H_2_O nanoparticles, produced by a sol–gel process, were used to decorate the GO through co-mixing in either ethanol or water. The green composites were pressed from these mixtures and the sintering conditions explored to ensure that graphene survived the processing. The sintering was conducted using a conventional controlled gas furnace, and the composites were characterized using X-ray diffraction, electron microscopy, and Raman spectroscopy.

## 2 Results and Discussion

### 2.1 Green Pellet Production

**Figure 1:**
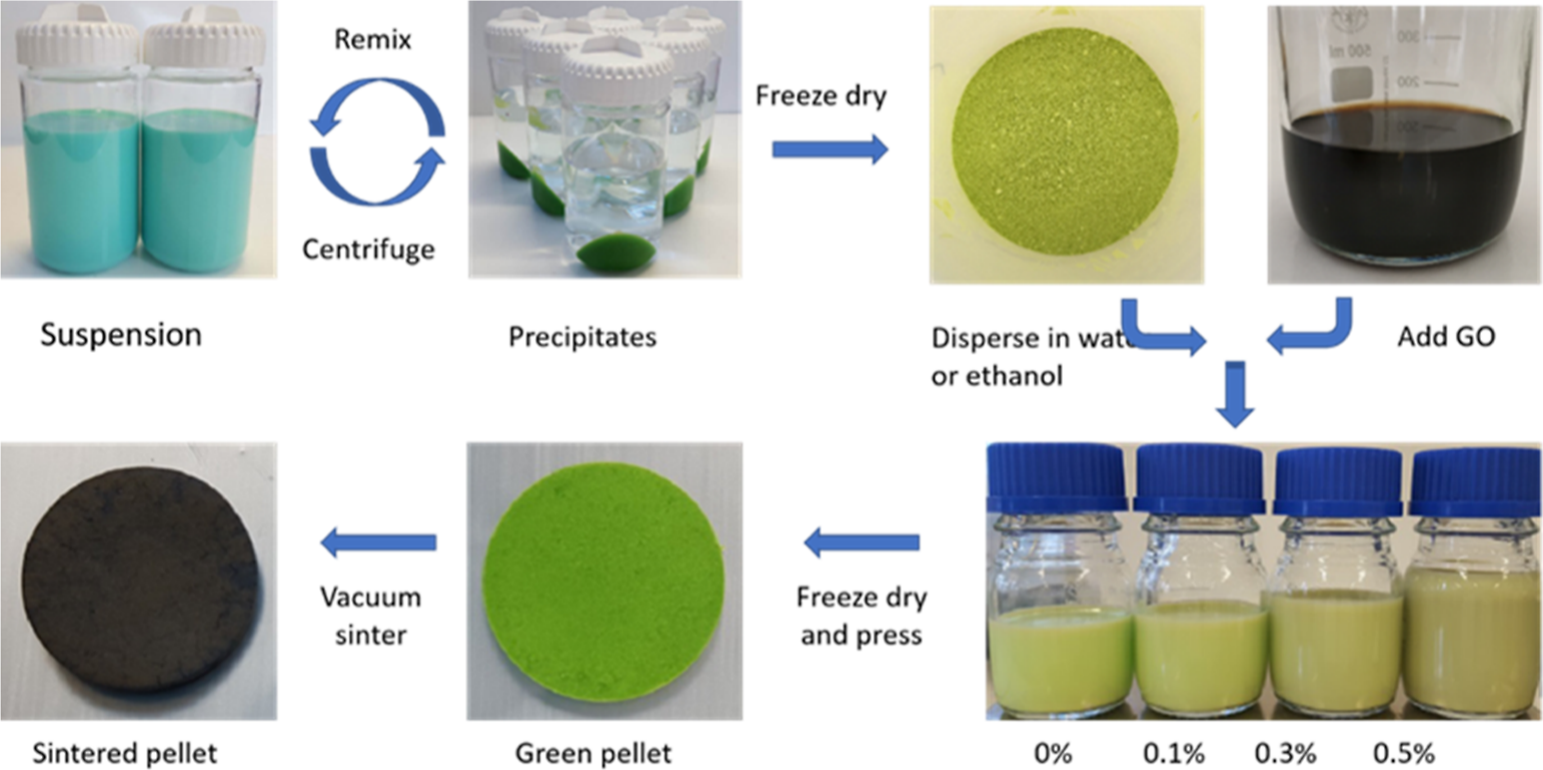
Synthesis route employed.

**Figure 2:**
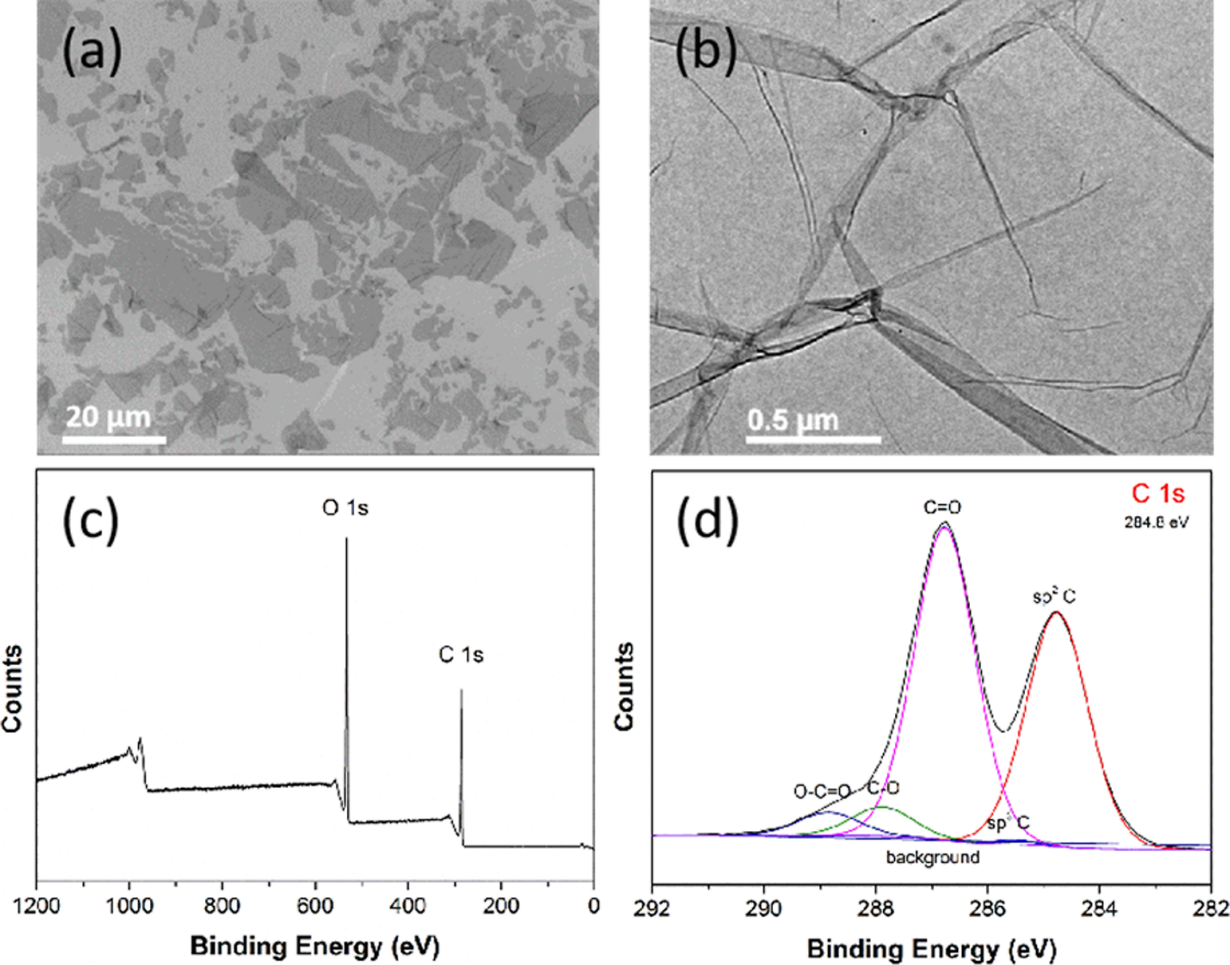
(a) SEM and (b) TEM images of GO, (c) survey XPS spectrum, and (d) C 1s XPS spectrum for GO.

**Figure 3:**
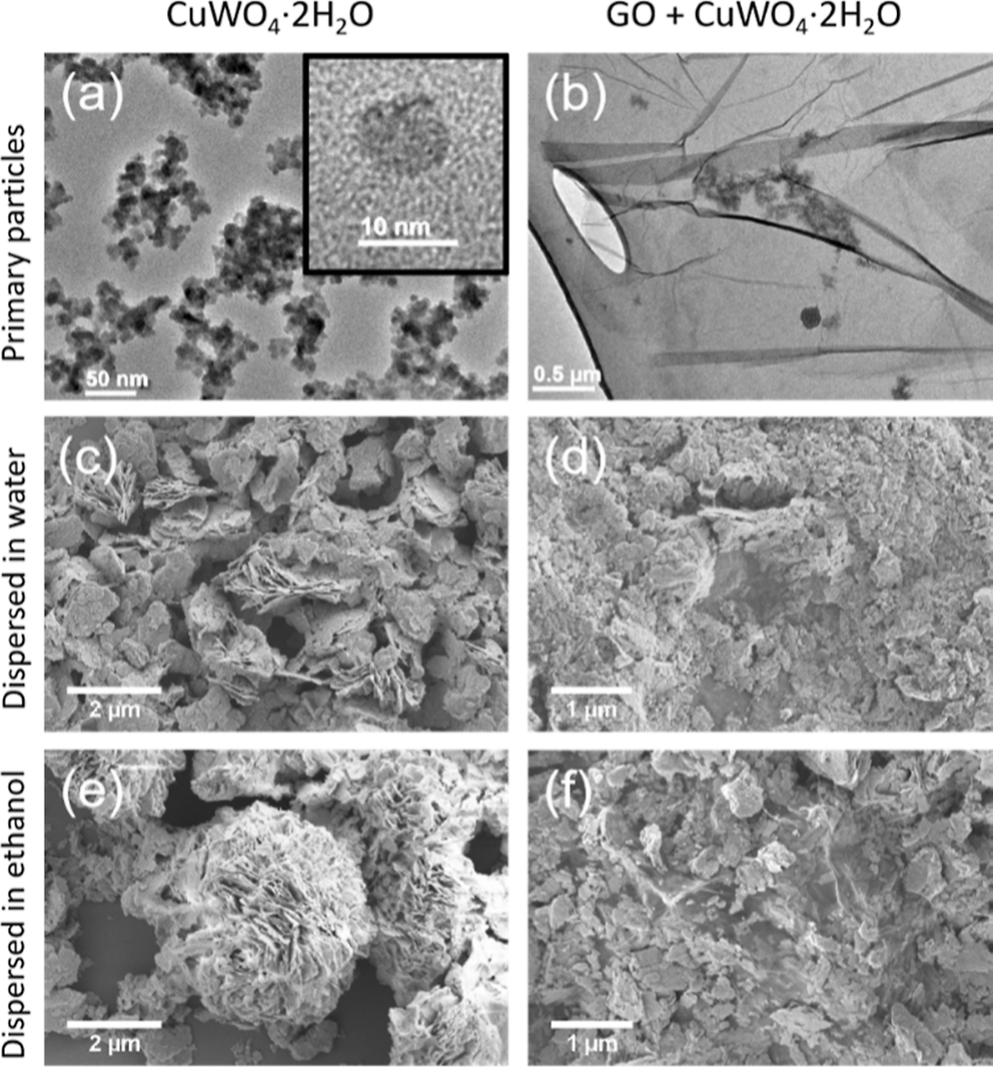
TEM of the (a) CuWO_4_·2H_2_O particles and (b) particles mixed with GO in water. (c–f) SEM of the control particles and particles mixed with GO from water and ethanol.

Figure 1 shows the route used to synthesize the sintered pellets. As is shown in Figure 2a,b, the GO produced from the Hummers method^24^ was found by electron microscopy to be predominantly monolayer, with some folding of the flakes. The diameters of the flakes were distributed bimodally with modes at 20 and 7.5 μm. The fully washed GO flakes had a zeta potential value of −41.6 ± 1.6 mV, meaning that they were stable in water. XPS determined that the C 1s and O 1s content was 72.4 and 27.6 at %, respectively, giving a C/O ratio of 2.62 (Figure 2c). The C 1s was initially calibrated to be at 284.8 eV and composed of sp^2^ C, sp^3^ C, C–O, C=O, and O–C=O with percentages of 39.12, 0.47, 51.62, 4.91, and 3.87%, respectively (Figure 2d). The CuWO_4_·2H_2_O nanoparticles were produced by the reaction of sodium tungstate dihydrate and copper sulfate pentahydrate followed by repeated washing of the precipitate with deionized water. Some of these precipitated particles were freeze-dried and analyzed by transmission electron microscopy (TEM). The particles were found to be spherical with a diameter of 8 nm (Figure 3a), which is comparable to the 10 nm reported by Souza et al.^25^

The CuWO_4_·2H_2_O nanoparticles and GO were dispersed together in the desired amounts in either deionized water or ethanol. (Two solvents were studied to establish if the solvent’s nature controls the decoration of the nanoparticles on the GO.) For all studies, a ratio of 1:1 by mass of CuWO_4_·2H_2_O to GO was used. In the case of aqueous dispersions containing 1 or 2 wt % GO/CuWO_4_·2H_2_O, it was found that the metal oxide particles settled over a period of 2 h, with the dispersion becoming greener at the bottom of the flask. The GO, however, remained in dispersion. When using ethanol as the dispersant, the GO settled preferentially, suggesting that 1 wt % GO was above its concentration limit in that solvent. The CuWO_4_·2H_2_O particles also displayed poor dispersability in the ethanol and settled rapidly.

The morphology and size of the resulting GO + CuWO_4_·2H_2_O mixtures and the control CuWO_4_·2H_2_O after vacuum drying at room temperature were analyzed using SEM. The control CuWO_4_·2H_2_O nanoparticles that had been dispersed in deionized water were found to be aggregated into platelets, which had stacked together (Figure 3c). The nanoparticles from the ethanol dispersion had also self-assembled into platelets, but these platelets were further aggregated into more complex structures (Figure 3e). The aggregation of these CuWO_4_·2H_2_O nanoparticles has been discussed previously for aqueous systems by Souza et al.,^25^ who suggested that the nanoparticles first self-assembled into rod-like structures which then aggregated into sheets and these sheets then formed “flower-like” nanocrystals. The platelets seen in this work from water are similar to Souza’s sheet structures, whereas the higher order flower-like crystals were seen in the ethanol system, possibly due to the lower dispersability in ethanol driving the aggregation process (Figure 3e).

We found using TEM that the CuWO_4_·2H_2_O nanoparticles decorated the GO sheets (Figure 3b) when GO was added to the CuWO_4_·2H_2_O dispersion. The CuWO_4_·2H_2_O particles had again assembled into platelets that were only observed decorating the GO (Figure 3d,f), implying that the self-assembly initiated on the GO flakes. The suggestion that the self-assembly process was affected by GO is further supported by the fact that the platelets on GO were less than half the length of those formed when no GO was present. Interestingly, GO prevented the flower-like aggregates being formed from the ethanol dispersion, perhaps due to the GO flakes being preferential nucleation sites compared to the platelets. It is worth comparing these structures with those obtained by Samantaray et al.^26^ who decorated rGO with CuWO_4_ through a hydrothermal route in which the CuWO_4_ was precipitated in the presence of GO. The direct precipitation method was found to produce a good degree of coverage as achieved here, but no self-assembly into higher order aggregates was observed.^26^ Overall, this evidence suggests that the primary 10 nm diameter particles form initially in the solution and then nucleate onto the GO flakes during drying.

**Figure 4:**
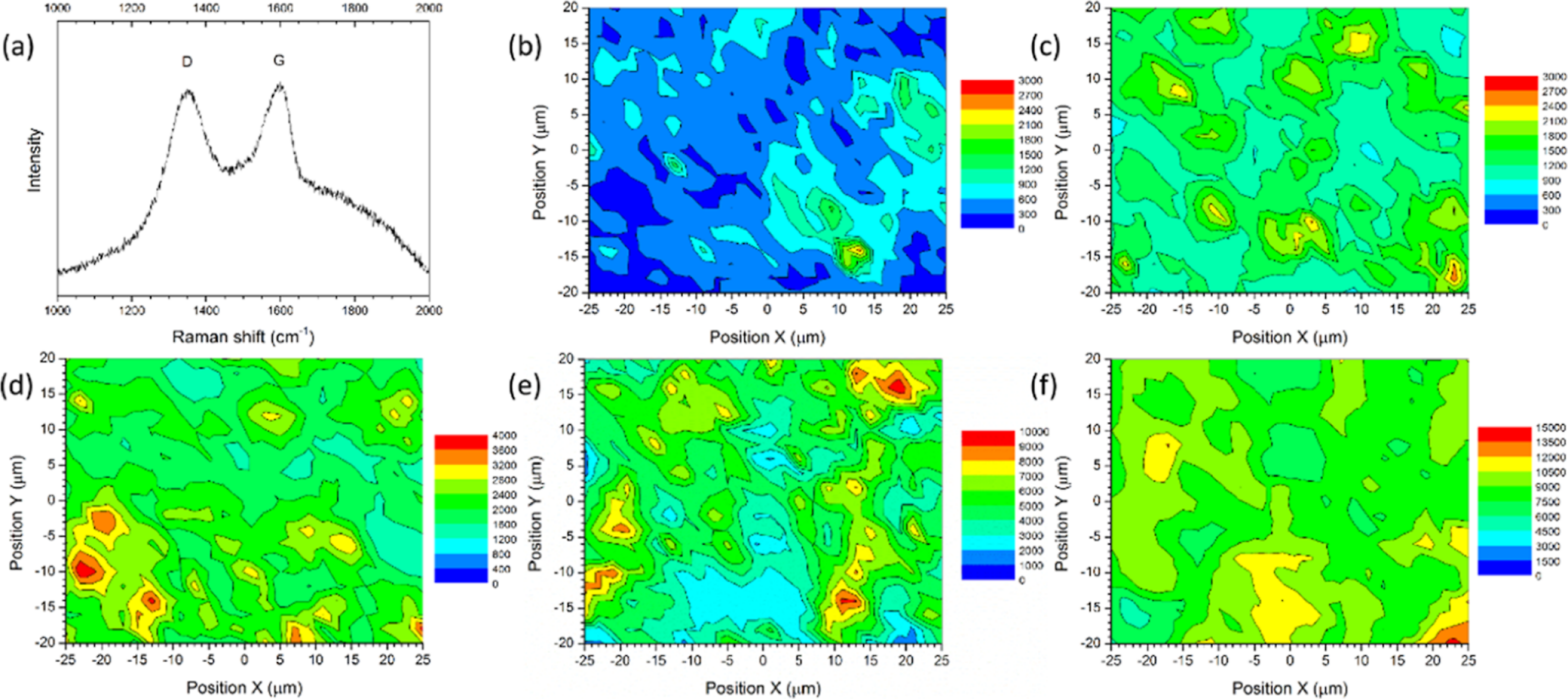
(a) Raman spectrum for GO in the compressed green pellet composite (CuWO_4_·2H_2_O-GO). Raman maps of the D band intensity for green composites with (b) 0.1, (c) 0.3, (d) 0.5, (e) 1, and (f) 2 wt % loadings of GO.

The coverage of the CuWO_4_·2H_2_O particles on the GO flakes was more uniform for those dispersed in water than those dispersed in ethanol. Thus, for the rest of this study, samples were produced by mixing the CuWO_4_·2H_2_O and GO in water. Green pellets were produced by compressing the freeze-dried GO + CuWO_4_·2H_2_O powders, and the dispersion of graphene within the pellets was analyzed using Raman spectroscopy mapping (Figure 4). GO has strong Raman D and G bands in the range between 1000 and 2000 cm^–1^, while the matrix has no bands within this range. Thus, the D band of GO was used as a distinguishing marker to assess the degree of dispersion of GO in the matrix, with spectra collected every 2 μm, using 1 μm spot size. A GO signal was found in 25% of the locations in the 0.1 wt % GO green composite. Whereas in the 1 wt % GO green composite, GO was found in 99% of the locations. Stronger GO bands and full GO coverage was observed for the 2 wt % composite.

### 2.2 Sintering

It is normally extremely difficult to sinter carbon nanoparticle-inorganic composites, particularly when oxides are present.^27^ Thus, a range of different sintering conditions (Table 1) were employed to determine which might be the most appropriate for the cold pressed, green pellets.

**Table 1:** Sintering Conditions for Each of the Four Conditions Used for the Green Pellets

	process			
condition no	ramp up	dehydration	reduction	ramp down
1	10 °C/min	1 h at 600 °C	1 h at 600 °C	slow
	2 mbar Ar	2 mbar Ar	0.5 mbar Ar	
	250 cm^3^/min	250 cm^3^/min	25 cm^3^/min	
2	10 °C/min		1 h at 1000 °C	rapid
	2 mbar Ar	N/A	0.5 mbar Ar	
	200 cm^3^/min		250 cm^3^/min	
3	10 °C/min	1 h at 600 °C	1 h at 600 °C	rapid
	2 mbar Ar	2 mbar Ar	1 mbar Ar	
	200 cm^3^/min	250 cm^3^/min	250 cm^3^/min	
4	10 °C/min	N/A	1 h at 600 °C	rapid
	2 mbar Ar		1 mbar Ar	
	200 cm^3^/min		250 cm^3^/min	

#### 2.2.1 Condition 1

**Figure 5:**
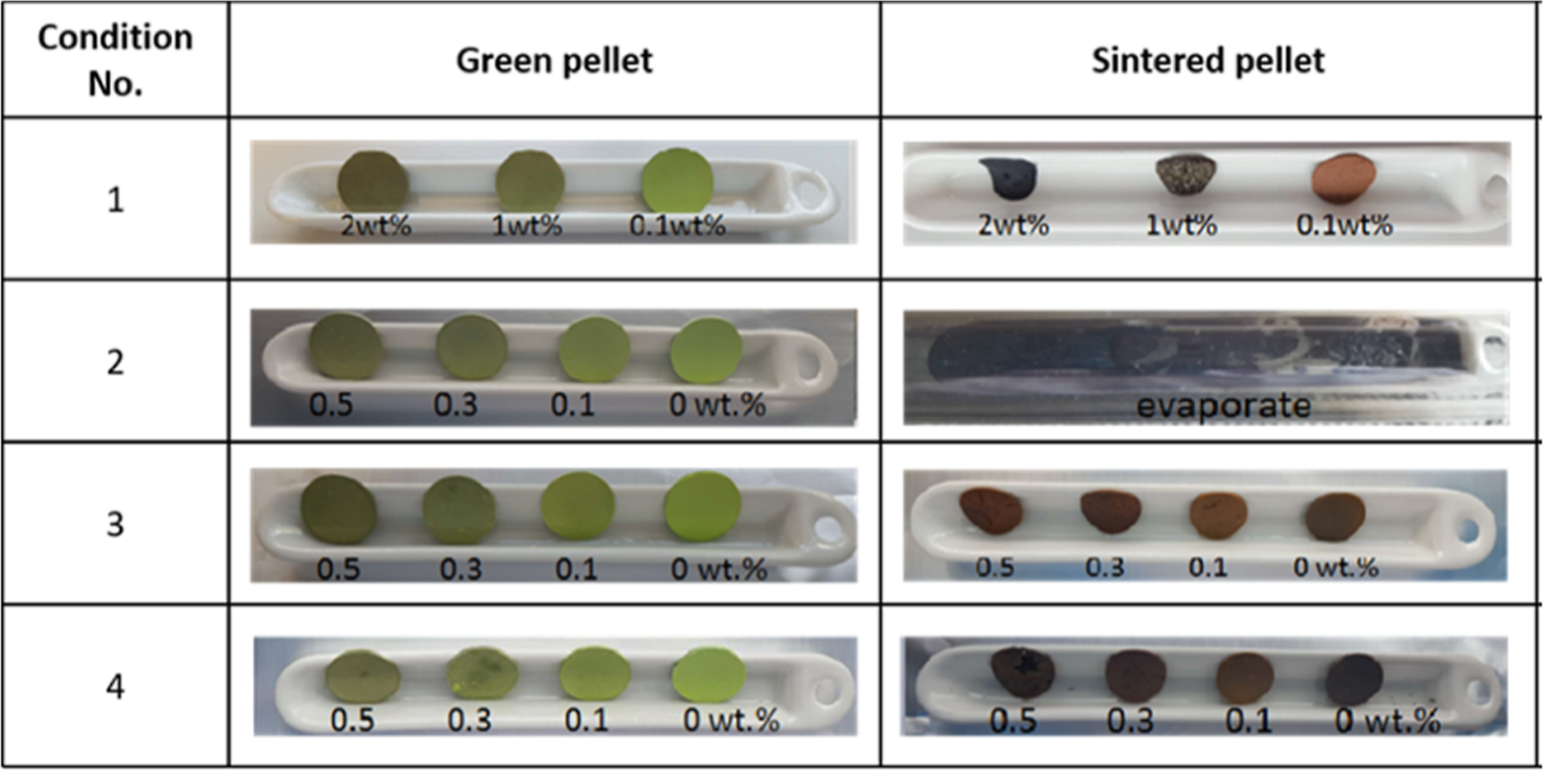
Photographs of the pellets before and after sintering. The green pellets were 13 mm in diameter.

In the first sintering process, pellets with loadings between 0.1 and 2 wt % GO were heated from room temperature to 600 °C at 10 °C/min and held at 1 h in an argon atmosphere flowing at a rate of 250 sccm (standard cubic centimeter per minute) at 1.8 mbar. The feed gas was then replaced with hydrogen at a rate of 25 sccm at 0.5 mbar, and the pellets were sintered for 1 h. The color of the sintered pellets changed significantly during the sintering, regardless of the GO loading (Figure 5). The loading of GO had a significant effect on the final sintered composites; the 2 wt % GO composite sintered directly to the alumina crucible, many blisters were visible on the surface of the 1 wt % GO composite, but the 0.1 wt % GO and control composites were relatively defect free.

**Figure 6:**
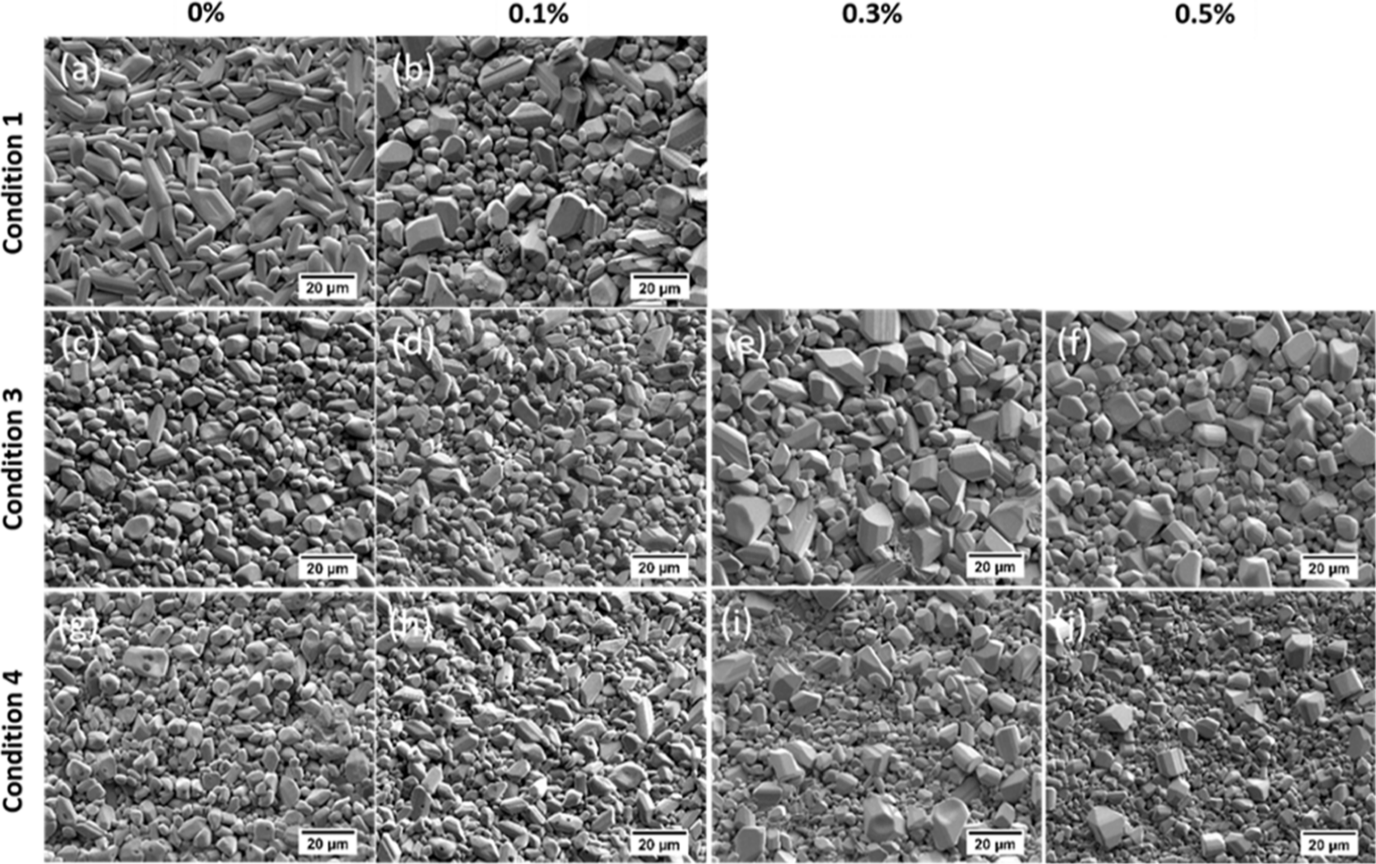
(a–j) SEM of the sintered composites with different loadings of GO (as given by the column heading) under different conditions (cf. Table 1). Note that under condition 1, samples containing 0.3 and 0.5% GO were not produced.

SEM of the control sample showed elongated grains with typical lengths of 10 μm and widths of 4 μm (Table 2, Figures 6a and S1a in the Supporting Information). Some pores were present, indicating that full densification was not achieved (Figure 6a).

**Table 2:** Grain Size Statistics (Mean Value and Standard Deviation) for Composites with Different GO Loadings under Different Sintering Conditions (Unit: μm)

	initial GO loadings (wt %)			
condition no	0	0.1	0.3	0.5
1	9.9 ± 3.6 (L)/3.9 ± 1.8 (S)	5.5 ± 3.3	N/A	N/A
2	N/A			
3	4.5 ± 1.9	4.4 ± 2.1	5.5 ± 2.7	4.7 ± 2.2
4	4.3 ± 2.0	4.2 ± 1.7	3.2 ± 2.1	2.9 ± 1.9

The morphology and the size of the sintered grains was found to change when the GO was added; the grains became polyhedral with an evenly split bimodal size distribution, with mode lengths of 11 μm and at 3 μm (Table 2, Figures 6b and S1b). This effect of GO on reducing grain size has been observed previously in cold-pressed aluminum where GO was found at grain boundaries.^23^

Energy-dispersive X-ray (EDX) was used to determine the chemical composition of two grain populations; the larger grains were found to have a Cu/W ratio of 1:1 and high content of oxygen (∼50 at. % oxygen), whereas the smaller grains were predominantly Cu (Cu/W ratio of 50:1) and were less oxidized (∼25 at. % oxygen).

#### 2.2.2 Condition 2

**Figure 7:**
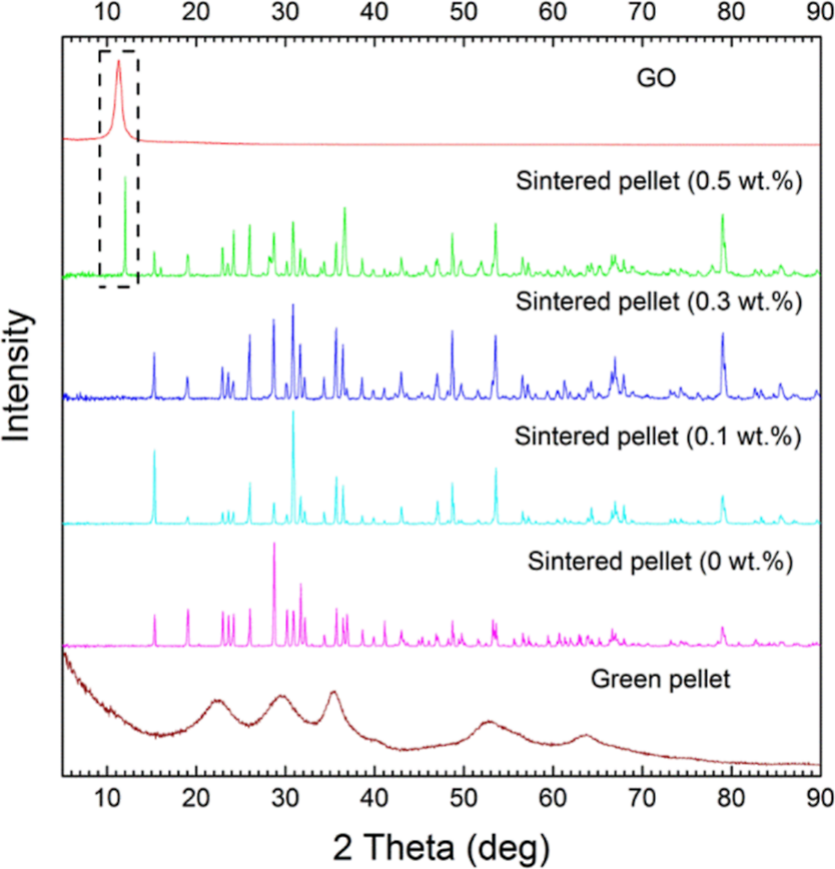
XRD of the GO, green pellets, and the sintered composites with different initial weight loading of GO. The XRD peaks of the sintered pellets are mainly from CuWO_4_.

In the second sintering run, a higher temperature of 1000 °C was used as the sintering temperature, and the hydrogen flow rate was increased with the aim of improving the density and the degree of reduction. The dehydration process was assumed to be completed before the end of the ramp-up phase. Hence, only a reducing atmosphere was used at the maximum temperature. Since pellets with loadings >1 wt % GO were not successfully sintered previously, lower loadings were used in condition 2 (0, 0.1, 0.3, and 0.5 wt %) (Figure 7). However, it was found that all samples evaporated under condition 2 as a result of the vacuum depressing the evaporation temperatures of the metals.

#### 2.2.3 Condition 3

To prevent the evaporation of the metal, the third sintering profile (Table 1) used a lower reduction temperature of 600 °C but with a higher hydrogen flow rate and a more rapid cooling rate.

The appearance of the sintered pellets was significantly better than that of the higher loading composites sintered at 600 °C under condition 1, confirming that there is a maximum loading of 0.5 wt % GO for successful sintering of the composites. The grains were found to be polyhedral with a diameter of 4.5 μm both with and without GO with EDX giving an oxygen content of typically 30 at. % (Figures 6c–f and S1 and Table 2). This association of a lower oxygen content with the smaller grains is consistent with the measurements in condition 1, where the smaller grains were the most reduced. This increased degree of reduction can be attributed to the high H_2_ flow rate used in condition 3 (250 sccm) compared to condition 1 (25 sccm). A few abnormal, elongated, and thin grains were also observed under condition 3. EDX showed that these grains contained residual sodium and sulfur from the starting precursor salts, implying that the precipitated CuWO_4_·2H_2_O nanoparticles were not sufficiently washed prior to freeze drying.

#### 2.2.4 Condition 4

The final sintering condition was based upon the successful condition 3 process, except for the removal of the dehydration step at 600 °C since it was believed that dehydration occurred during the ramping phase. This decrease in the total time spent at higher temperatures resulted in same grain size as that found in condition 3 (Figures 6g–j and S1 and Table 2), but the oxygen content was also higher in the grains, typically at around 50 at. %. X-ray diffraction (XRD) showed that the material had not been reduced, despite using a higher temperature than Samantaray et al.^26^ to sinter their copper tungstate.

XRD can be used to follow the phase structure of the materials during sintering. The XRD trace for the green pellet in Figure 7 is identical to that of the monoclinic structure of CuWO_4_·2H_2_O reported by Souza et al.^25^ The XRD traces for the sintered pellets are quite different and similar to those of the triclinic form of CuWO_4_ also reported by Souza et al. who showed that heating above 400 °C led to the loss of water and a transformation to this new phase. More detailed analysis of the XRD traces showed that although the predominating phase is copper tungstate, Bragg reflections corresponding to CuO and Cu_2_O are also present (Figure S2 in the Supporting Information). The (002) GO reflection can also be clearly seen in the sintered 0.5 wt % GO composite in Figure 7, implying that aggregated, stacked GO was still present after the high-temperature processing. The reflection had, though, moved from 11.3 to 12.1° degrees and sharpened relative to the as-made GO. This change in position and shape was due to the partial reduction of GO during sintering.^28,29^ The (002) reflection was absent in the composites sintered with <0.5 wt % GO, which could be due to either a better dispersion of GO at low loadings or insufficient GO to give diffraction peaks compared to the strongly diffracting metal and metal oxide particles.

**Figure 8:**
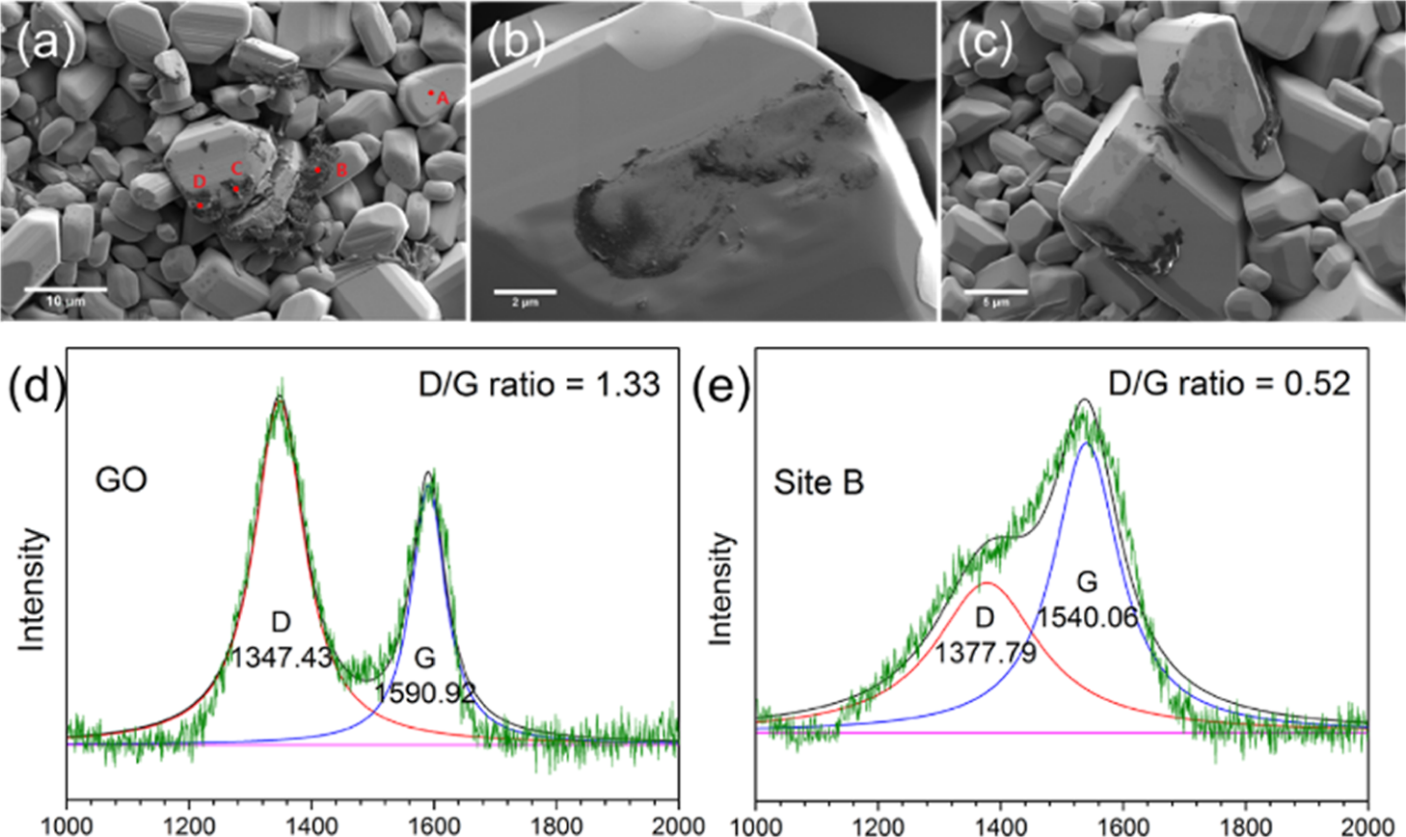
(a,b) SEM images taken from a composite with 0.5 wt % GO under condition 3, (c) SEM image for composite with a 0.3 wt % GO sintered under condition 4. (d) Typical Raman spectrum from a green pellet with 0.5 wt % GO (before sintering) in Figure 7. (e) Typical Raman spectrum from a pellet with 0.5 wt % after sintering.

SEM showed that some of the GO was lost during sintering, probably due to oxidation (Figure 8). The GO flakes that remained were aggregated at the edges of the grains, and their carbonaceous nature was confirmed by EDX obtained at 5 kV (Figure 7a and Table S1), with the regions B, C, and D containing more carbon than grain A.

Raman spectra were also collected from the four regions indicated in Figure 8a. The GO flakes in the composite are significantly different before and after sintering (Figure 8). Before sintering, the D and G bands (shown in Figure 8d as a typical example) for GO were centered at 1347.4 and 1590.9 cm^–1^, respectively, with a D/G intensity ratio of 1.33. After sintering, the D and G bands for GO shifted and merged, i.e., the D band shifted up to 1375 cm^–1^ and G band shifted down to 1540 cm^–1^ (shown in Figure 8e). In addition, the D/G intensity ratio decreased to 0.52, indicating fewer defects. Similar sintered spectra were seen in locations C and D. This significant shift of the D and G peaks toward each other is higher than that expected for the simple reduction of GO. Comparison of these spectra with those from carbon films grown by Wang et al. on a titanium substrate sputtered in a H_2_/CH_4_ atmosphere strongly suggests that the GO became hydrogenated during the sintering process in the high-temperature hydrogen atmosphere.^30^ Indeed, such hydrogenation would explain the relatively intense (002) peak still observed in the X-ray diffraction pattern, despite the high heat treatments used.

## 3 Conclusions

We have been successful in our aim to produce precursors of Cu–W-rGO composites. GO was decorated by 8 nm diameter CuWO_4_·2H_2_O nanoparticles by mixing GO with nanoparticles produced by a precipitation process. The nanoparticles were found to self-assemble into platelets and flower-like-structures when dried from water and ethanol, respectively. The addition of GO led to the growth of the platelets from the GO’s surface regardless of solvent, showing the good interaction of GO with CuWO_4_·2H_2_O. This strong interaction also prevented the higher order flower-like aggregates from growing. Raman spectroscopy mapping showed that GO was evenly distributed in the green pellets made from these materials. The degree of GO loading was found to influence the sinterability of the composites, with the maximum loading established as 0.5 wt %. It was found that 1000 °C was too a high a temperature to sinter the composites, with significant evaporation of the metals occurring. A temperature of 600 °C, however, produced coherent sintered pellets, with a mixture of metals and metal oxides present. Under slow cooling, the addition of GO changed the grain morphology from a needle-like structure to a polyhedral shape. The grain size was also found to be dependent on the extent of the oxidization, with the more reduced grains being smaller in diameter. GO was confirmed to have survived the sintering process; however, Raman spectroscopy showed that rather than being reduced, the GO possibly became hydrogenated due to the presence of a hydrogen atmosphere. Future work will use SPS to reduce the pellets fully and then study the physical properties of these composites such as their arc erosion resistance.

## 4 Experimental Section

### 4.1 Materials

Sodium tungstate dihydrate (99%) was purchased from Fluorochem Limited. Copper sulfate pentahydrate (>99%) was obtained from the Scientific Laboratory Supplier. GO suspensions were synthesized using the modified Hummers method,^24^ incorporating a dialysis step. The concentration of the as-produced GO suspensions was measured to be 8.8 mg/mL. All reagents were of analytical grade and used as received without further purification.

### 4.2 Methods

A 0.25 M solution of sodium tungstate dihydrate and a 0.25 M solution of copper sulfate pentahydrate in deionized water were prepared. 50 mL of each of these solutions were then mixed together and stirred for 1 h using a magnetic stirrer at room temperature. CuWO_4_·2H_2_O precipitates were produced during this hour. These precipitates were purified by centrifugation at a speed of 5000–10,000 rpm/min for 10–30 min. After removing the supernatant, the precipitates were redispersed in deionized water with the aid of magnetic stirring. The purification by centrifugation step was then repeated several times to increase the final purity.

To decorate the GO, 0.5 g of the washed precipitate was then added to either deionized water or ethanol, to which the required amount of GO relative to the weight of nanoparticles was then added. The dispersion was then left for 2 h, after which the water/ethanol supernatant was discarded and the resulting powder freeze-dried. The composite samples were prepared by cold pressing 0.3 g of the freeze-dried GO + CuWO_4_·2H_2_O powders into a pellet under 2 tons force for 1 min using a manual hydraulic press and a 13 mm diameter die. Pellets using pure CuWO_4_·2H_2_O powder were also produced under the same conditions. These compacted pellets were loaded onto an alumina crucible boat and inserted into a tube furnace to be sintered under vacuum. Four different sintering conditions were used as outlined in Table 2.

### 4.3 Characterization

The GO, powders, and sintered composites were studied using scanning electron microscopy (Zeiss Ultra 55 FEG-SEM, Zeiss Sigma FEG-SEM) and transmission electron microscopy (FEI Tecnai TF30 FEG-TEM). X-ray photoelectron spectroscopy (XPS) was used to study the chemical nature of GO. Freeze-dried GO powders were prepared for the XPS experiment. XPS was performed using a Kratos Axis Ultra DLD equipped with a monochromatic Al Kα X-ray source (1486.6 eV, 10 mA emission, 15 kV) and a charge neutralizer to remove the effects of differential charging. Survey spectra used an electron energy analyzer pass energy of 80 eV, whereas the high-resolution spectra used 20 eV. The X-ray beam size was 300 × 700 μm. Experiments were carried out at ultrahigh vacuum, and spectral analysis was then performed using CasaXPS.

Raman spectroscopy (Renishaw inVia Raman System, using 633 nm HeNe excitation laser) was used to identify the GO and determine the degree of dispersion of the GO in the composite. XRD experiments were conducted to investigate the structure and phase identification for the composite. The equipment used was a PANalytical X’Pert Pro X’Celerator X-ray diffractometer with Cu Kα (λ = 0.15418 nm).

## Supporting Information Available

Grain size distribution plots for samples with different GO loadings under different conditions; XRD patterns for the sintered pellet (0% GO) with CuWO_4_, CuO, WO_3_, W, Cu, and Cu_2_O; and elemental compositions obtained from EDS results for the sites in Figure 8a (PDF)
