# Supplementary material for: Production of Graphene/Inorganic Matrix Composites through the Sintering of Graphene Oxide Flakes Decorated with CuWO4·2H2O Nanoparticles
Source: ACS Omega. 2023 Apr 2;8(14):13131–9. doi: 10.1021/acsomega.3c00063 (PMC10099426; doi:10.1021/acsomega.3c00063)
Supplement: Supplementary file 1 — ao3c00063_si_001.pdf [file ao3c00063_si_001.pdf]

Supporting Information

The Production of Graphene/Inorganic-Matrix Composites through the Sintering of Graphene Oxide Flakes Decorated with $CuWO_4 \cdot 2H_2O$ Nanoparticles

Fei Lin[a], Yuzhen Zhou[b], Ruoyu Xu[b], Mingyu Zhou[b], Andrew M Connolly[a], Robert J Young[a] and Ian A Kinloch[a]*

[a] Department of Materials and the National Graphene Institute, University of Manchester, Oxford Road, Manchester, UK

[b] Department of Electrical Equipment & Material, Global Energy Interconnection Research Institute Europe GmbH, Berlin, Germany

* Corresponding author: ian.kinloch@manchester.ac.uk

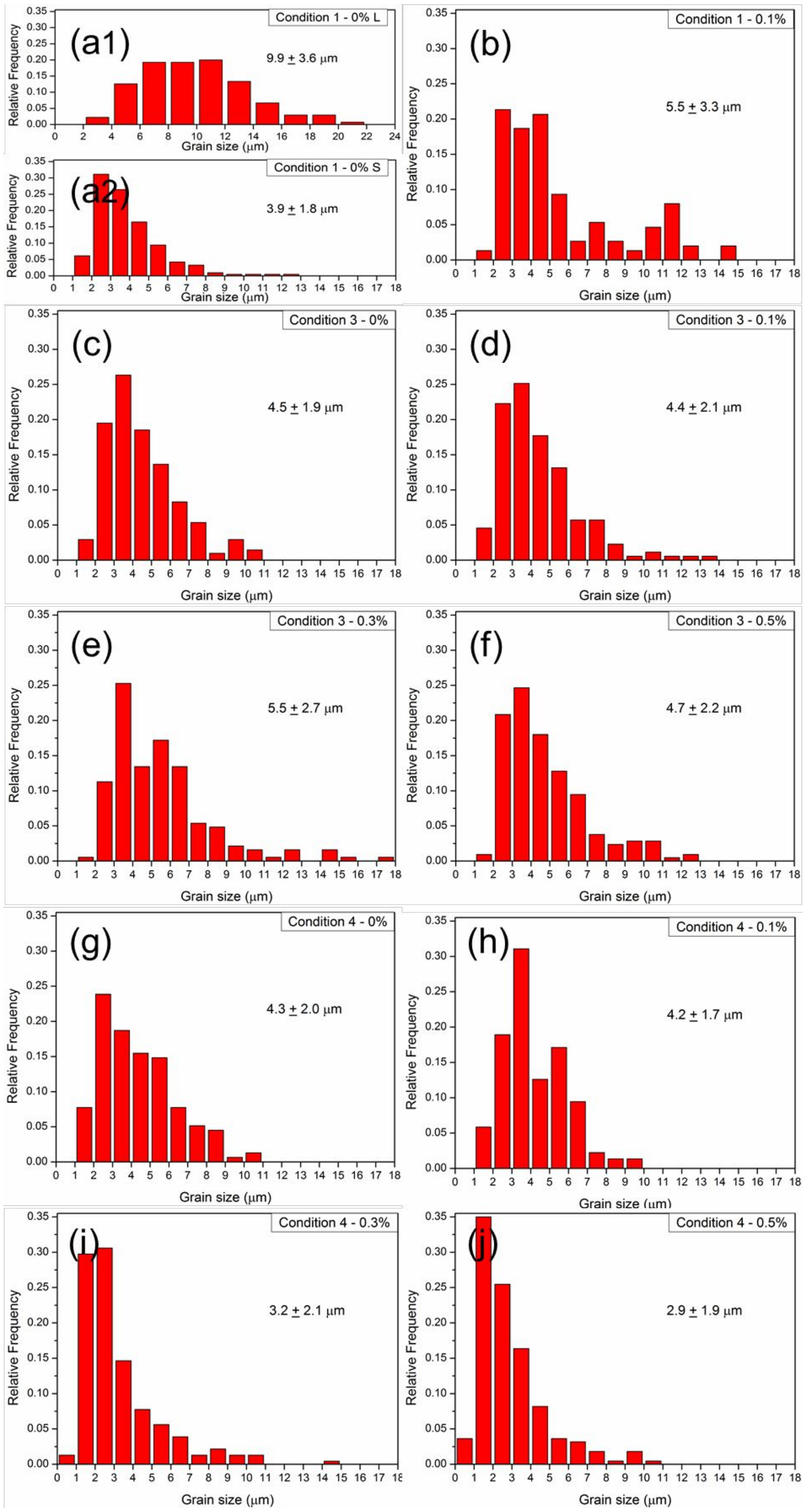

Figure S1: Grain size distribution plots for samples with different GO loadings processed under different conditions. Note: (a1) (a2) measured the grains in the long and short axis respectively. (b-j) measured the grains in the long direction (x axis). All dimensions are in microns.

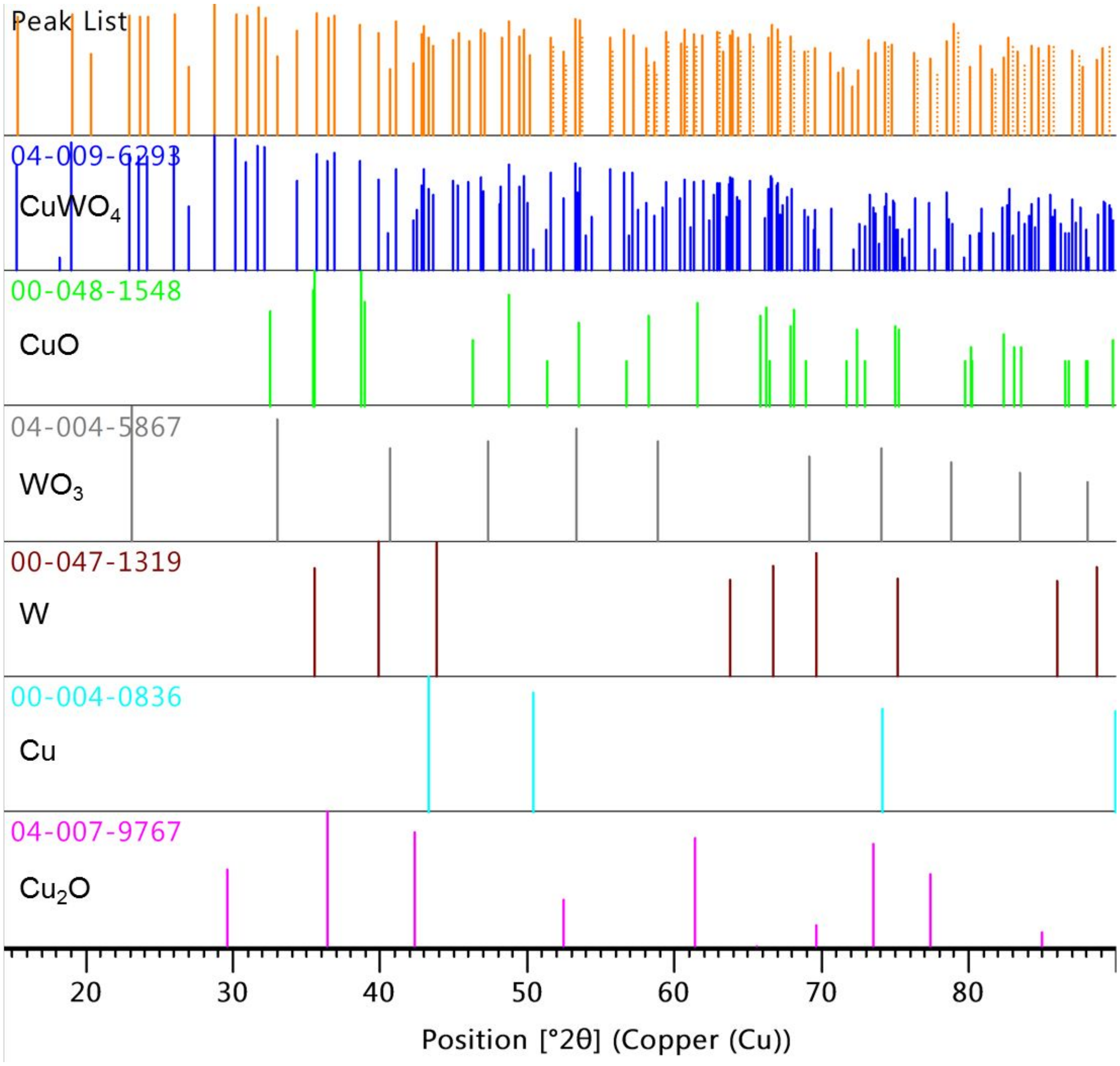

Figure S2: XRD patterns for the sintered pellet (0% GO) and $CuWO_4$, CuO, WO_3, W, Cu and Cu_2O.

Table S1: Elemental compositions obtained from EDS results for the sites in Fig. 8a.

**Element**	**Site**			
**(wt.%)**	A	B	C	D
**C K**	15	81	26	59
**O K**	16	17	18	20
**Cu L**	17	<0.5	13	6
**W M**	51	2	43	15
